# Simultaneous common bile duct clearance and laparoscopic cholecystectomy: experience of a one-stage approach

**DOI:** 10.1007/s00068-018-0921-z

**Published:** 2018-02-07

**Authors:** Shahin Mohseni, John Ivarsson, Rebecka Ahl, Sinan Dogan, Sten Saar, Arvo Reinsoo, Teesi Sepp, Karl-Gunnar Isand, Edvard Garder, Ilmar Kaur, Heiti Ruus, Peep Talving

**Affiliations:** 1 Division of Trauma and Emergency Surgery, Department of Surgery, Orebro University Hospital, 701 85 Orebro, Sweden; 2 Division of Acute Care Surgery, Department of Surgery, North Estonia Medical Center, 13412 Tallin, Estonia; 3 Orebro University, Fakultetsgatan 1, 702 81 Orebro, Sweden; 4 Department of Surgery, Karolinska University Hospital, 171 76 Stockholm, Sweden

**Keywords:** Laparoscopic cholecystectomy, ERCP, One-stage approach

## Abstract

**Introduction:**

The timing and optimal method for common bile duct (CBD) clearance and laparoscopic cholecystectomy remains controversial. Several different approaches are available in clinical practice. The current study presents the experience of two European hospitals of simultaneous laparoscopic cholecystectomy (LC) and intra-operative endoscopic retrograde cholangiopacreatography (IO-ERCP) done by surgeons.

**Methods:**

Retrospective analysis of all consecutive patients subjected to LC + IO-ERCP during their index admission between 4/2014 and 9/2016. Data accrued included patient demographics, laboratory markers, operation time (min) reported as mean (± SD) and hospital length of stay (LOS) reported as median (lower quartile, upper quartile).

**Results:**

During the 29-month study, a total of 201 consecutive LC + IO-ERCPs were performed. The mean age of patients was 55 ± 19 years and 67% were female. The mean intervention time was 105 ± 44 min. The total LOS was 4 (3,  7) days and the post-operative LOS was 2 (1,  3) days. A total of 6 (3%) patients experienced post-interventional pancreatitis and two (1%) patients suffered a Strasberg type A bile leak. All patients were successfully discharged.

**Conclusion:**

Simultaneous LC + IO-ERCP is associated with few complications. Further studies investigating cost-benefit and patient satisfaction are warranted.

## Background

Common bile duct (CBD) stones are encountered in up to 20% of patients who present with biliary colic or acute cholecystitis requiring urgent cholecystectomy [1, 2]. The timing and management of CBD stones in these settings is a matter of debate. Advances in laparoscopic surgery have made laparoscopic CBD exploration a viable alternative to an open CBD exploration [3]. However, since its introduction in the late 1970s, [4] endoscopic retrograde cholangiopancreatography (ERCP) has widely replaced the surgical approach for CBD stone clearance [5].

With ERCP evolving capabilities in the management of CBD stones, most patients are currently managed in a two-stage procedure with pre- or postoperative ERCP and laparoscopic cholecystectomy (LC). These algorithms  frequently result in multiple admissions and extended hospital length of stay (HLOS) [6]. Promising results including fewer complications, shorter HLOS and reduced overall cost have been noted with a one-stage procedure of LC + intraoperative (IO) ERCP [6–9]. We sought to report our experiences from two hospitals in Northern Europe using a one-stage procedure (LC + IO-ERCP) for management of cholelithiasis with suspected CBD stones. At both study sites, the IO-ERCP is performed by surgical teams with two different approaches; the transcystic “rendezvous” approach using guidewire guidance and the traditional intraoperative ERCP technique. The purpose of the study was to determine the incidence of procedural complications following the one-stage IO-ERCP. In addition, we aimed to compare the one-stage approach to the historical two-staged management. Also, we aimed to compare the alternative approaches of CBD clearance in our study sites.

## Methods

After IRB approval, all consecutive patients admitted to Orebro University Hospital (OUH) in Sweden and to North Estonia Medical Center (NEMC) in Estonia subjected to LC + IO-ERCP (NOMESCO procedure codes: JKA21, TJK01, UJK02, JKE02, JKE12) during their index admission between 4/2014 and 9/2016 were included. Data accrued included patient demographics, laboratory markers, American Association of Anesthesiologists (ASA) classification score, Charlson’s co-morbidity index (CCI), pre-operative diagnosis, mode of CBD stone imaging, operative time (min.), complications, total and post-operative hospital length of stay (LOS). Categorical variables are reported as percentages and continuous variables are reported as mean ± standard deviation (SD) or median (lower quartile (LQ), upper quartile (UQ)).

The primary outcome of the study was the overall incidence of ERCP-related complications. Secondary outcomes included procedural complications between the two different ERCP approaches as well as postoperative and total hospital length of stay.

All statistical analyses were performed using SPPS for Windows version 17 (SPSS, Chicago, IL, USA).

### The surgical methods

**Fig. 1:**
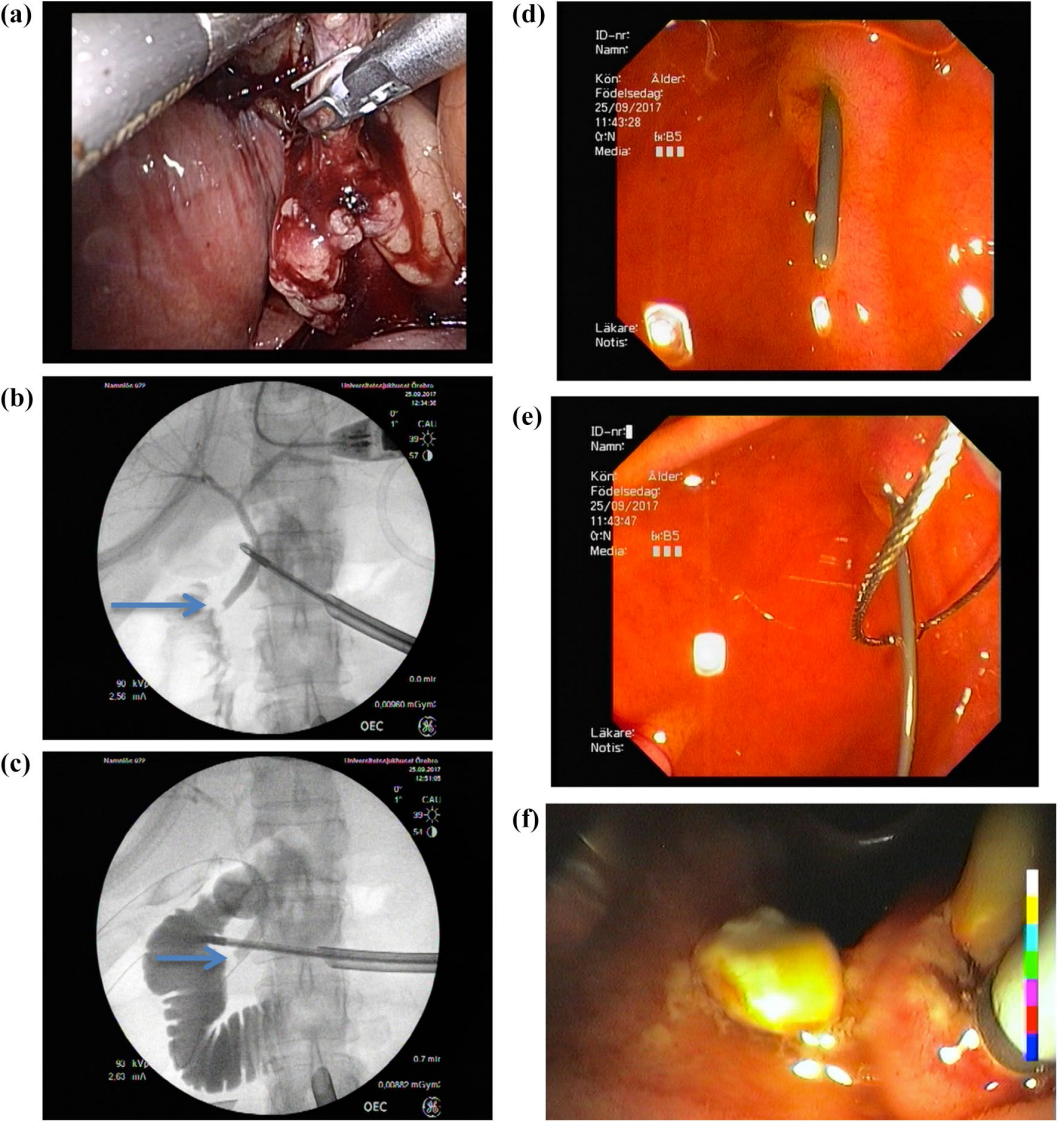
ERCP with Rendezvous technique: **a** cholangiography tube in the cystic duct; **b** cholangiography confirming distal CBD stone (arrow); **c** cholangiography with guidewire in place; **d** guidwire in place (Ampulla of Vater); **e** guidwire caught by endoscopist; **f** extraction of CBD stones by balloon clearance

Fellowship-trained surgeons performed all of the IO-ERCPs at the study sites. At Orebro University Hospital (OUH) there are four upper gastrointestinal surgeons providing ERCP service to acute care surgery, whereas at the North Estonia Medical Center (NEMC) acute care surgery providers perform all laparoscopic cholecystectomies and IO-ERCPs. Slight differences in management of suspected CBD stones existed in the involved facilities providing opportunities for comparison. At OUH, the LC + IO-ERCP is initiated with a laparoscopic cholecystectomy. After surgical identification of Calot’s triangle, a surgical clip is placed on the cystic duct in proximity to the neck of the gall bladder. A small incision is made into the cystic duct allowing the performance of an intraoperative cholangiogram (Fig. 1a, b). When a CBD stone is identified, a guidewire is passed through the cystic duct incision into the duodenum through the Ampulla of Vater (Fig. 1c). The pneumoperitoneum is deflated and endoscopy is performed. The guidewire is looped in a “rendezvous” fashion by the surgeon performing endoscopy (Fig. 1d, e). This maneuver is followed by a papillotomy and the CBD clearance using a balloon (Boston Scientific, MA, USA) (Fig. 1f). The entire procedure is performed with the patient in a supine position. Following ERCP, the duodenum and the stomach are deflated and the laparoscopic cholecystectomy is completed. Alternatively, at the NEMC, the decision for simultaneous procedure is defined by the preoperative risk stratification based on history, imaging, and laboratory markers. When a CBD stone is suspected, a laparoscopic cholecystectomy is performed in a standard fashion followed by intraoperative ERCP. The ERCP-facilitated CBD clearance includes cannulation of the Ampulla of Vater followed by cholangiogram, papillotomy and balloon or basket clearance of the CBD when stones are identified. When CBD clearance is not feasible, insertion of 1–2 plastic CBD stents (Boston Scientific Corp., MA, USA) are performed. In these instances, a follow-up ERCP is scheduled in 4 weeks’ time for CBD clearance and stent removal.

## Results

During the 29-month study period, a total of 201 patients were subjected to simultaneous LC + IO-ERCP during their index admission. A total of 107 cases and 94 cases were treated at NEMC and OUH, respectively. The mean age of the cohort was 55 (± 19) years, 67% were females, with an ASA classification [median (LQ, UQ)] score of 2 (1, 2), and the CCI [median (LQ, UQ)] was 2 (1, 4) (Table 1). Depicted in Table 1 are the preoperative laboratory test results and diagnoses. All patients had preoperatively ultrasound verified gallstones in the GB and/or in the CBD. The preoperative diagnosis was cholecystolithiasis in 93 (46%) patients, acute cholecystitis in 61 (30%) patients, gallstone pancreatitis in 40 (20%) and cholangitis in 9 (5%) patients. CBD stones were detected in a total of 175 (87%) cases preoperatively (Table 1). The preoperative imaging modality for CBD stones included ultrasound in 92 (46%), MRCP in 46 (23%) and CT in 36 (18%) cases. Twenty-four (26%) patients at OUH did not have a radiologically verified CBD stone prior to surgery and these were identified by routine intraoperative cholangiography (Table 1).

**Table 1:** Patient demographics, laboratory, preoperative diagnosis and modality for CBD diagnosis

	Total	OUH	NEMC	*p* value
Total number of patients, *n*	201	94	107	
**Patient demographics**				
Female gender, n (%)	134 (67%)	59 (63%)	75 (70%)	0.27
Age (mean ± SD)	55 ± 19	51 ± 35	58 ± 20	0.05
ASA class (median, [LQ, UQ])	2 [1, 2]	2 [1, 2]	N/A	−
CCI score (median, [LQ, UQ])	2 [1, 4]	2 [1, 4]	3 [1, 4]	0.05
**Laboratory markers**				
White blood count, 10^9^/L (mean ± SD)	9.4 ± 4.1	9.6 ± 4.6	9.1 ± 3.6	0.40
CRP, mg/L (mean ± SD)	41 ± 71	36 ± 72	45 ± 70	0.40
AST, µkat/L (mean ± SD)	4.3 ± 3.8	5.3 ± 4.2	3.4 ± 3.1	0.001
ALP, µkat/L (mean ± SD)	3.5 ± 2.3	3.4 ± 2.1	3.6 ± 2.5	0.52
Bilirubin, µmol/L (mean ± SD)	54 ± 39	51 ± 35	56 ± 42	0.30
Lipase, µkat/L (mean ± SD)	14 ± 24	16 ± 25	10 ± 22	0.12
**Preoperative diagnosis**				
Cholecystolithiasis, *n* (%)	93 (46%)	81 (86%)	12 (11%)	0.17
Acute cholecystitis, *n* (%)	61 (30%)	36 (38%)	25 (23%)	< 0.001
Gallstone pancreatitis, *n* (%)	40 (20%)	23 (25%)	17 (16%)	< 0.001
Cholangitis, *n* (%)	9 (5%)	5 (5%)	4 (4%)	0.74**
**Verified preoperative CBD stone**				
Verified CBD stone, *n* (%)	177 (88%)	70 (74%)	107 (98%)	< 0.001
Not verified CBD stone, *n* (%)	24 (12%)	24 (26%)	0	–
**Modality for CBD stone verification**				
Ultrasound, *n* (%)	92 (46%)	16 (17%)	78 (73%)	
MRCP, *n* (%)	46 (23%)	46 (49%)	N/A	
CT, *n* (%)	36 (18%)	7 (7%)	29 (27%)	
CBDS confirmed on IOC, *n* (%)	94 (47%)	94(100%)	N/A	

*LQ* lower quartile, *UQ* upper quartile, *CCI* Charleson’s comorbidity index, *CBD* common bile duct, *MRCP* magnetic resonance cholangio-pancreatography, *CT* computer tomography, *IOC* intraoperative cholangiography

**Fisher exat test, two sided *p* value

Only 8 (4.0%) patients experienced procedure-related complications; 6 (3%) suffered post-interventional pancreatitis and 2 (1%) demonstrated a Strasberg type A bile leak (Table 2).

**Table 2:** Outcomes

	Total	OUH	NEMC	*p* value
Surgery time, min (mean ± SD)	105 ± 44	120 ± 43	91 ± 41	< 0.001
Iatrogenic pancreatitis, *n* (%)	6 (3%)	4 (4%)	2 (2%)	0.42*
Bile leak, *n* (%)	2 (1%)	2 (2%)	0 (0%)	0.22*
Hospital LOS, days (median, LQ, UQ])	4 [3, 7]	4 [3, 7]	4 [3, 6]	0.42
pLOS, days (median, [LQ, UQ])	2 [1, 3]	1.5 [1, 3]	2 [1, 4]	0.04

*LQ* lower quartile, *UQ* upper quartile, *LOS* length of stay, *pLOS* postoperative length of stay

*Fisher exat test, two sided *p* value

The mean operative time was 105 (± 44) min. NEMC had a significantly shorter operative time compared to OUH [91 (± 41) vs. 120 (± 43) min, *p* < 0.001] due to the institutional practice to perform intra-operative cholangiography in all cholecystectomy cases at the OUH. Median (LQ, UQ) LOS after surgery was 2 (1, 3) days and the total length of stay was 4 (3, 7) days. There were no differences in the length of stay between the two institutions (Table 2).

## Discussion

Concomitant CBD stones are encountered in up to 18% of patients undergoing acute cholecystectomy for gall stone complications [10]. With advancements in laparoscopic techniques and acute care surgery capabilities, the management in these instances has evolved from open CBD exploration to laparoscopic techniques of CBD clearance and cholecystectomy. The one-stage laparoscopic approach, which entails a transcystic or a transductal intervention in the management of choledocholethiasis, has been advocated by several authors. However, there is a declining trend in CBD exploration since this approach is both  technically demanding and time-consumingwhich  tips the balance in favor of endoscopic CBD clearance using ERCP [5, 11]. ERCP with cholangiography and papillotomy has been available in most referral centers for more than three decades [12]. The overall success rate of ERCP in terms of CBD clearance by an experienced endoscopist is over 95% [5, 13]. ERCP is applied as part of a one-stage (laparoscopic cholecystectomy + IO-ERCP) or as a two-stage procedure (pre- or post-operative ERCP and LC). There are several advantages with a one-stage compared to a two-stage approach. Selecting patients with CBD stone(s) for ERCP can be challenging even with the use of different predictive models based on clinical, laboratory and imaging findings [14, 15]. This makes the one-stage approach more appealing when intra-operative cholangiography is available. A total of 24 (12%) patients in the currents study had CBD stones detected by intraoperative cholangiography that had not been identified preoperatively.

There are several ERCP procedure-related complications including pancreatitis (1–30%), pancreatic necrosis (0.3–0.6%) and mortality (0.4%), that can be avoided with over the guidewire (the Rendezvous technique) IO-ERCP. This allows avoidance of the critical phase of retrograde inadvertent cannulation of the pancreatic duct [15]. A meta-analysis done by Arrezo and colleagues including a total of 430 patients from four randomized controlled trials observed a lower incidence of overall complications for the one-stage Rendezvous technique (11.2%) compared to the two-stage approach (18.1%) [OR 0.56; 95% confidence interval (CI) 0.32–0.99; *p* = 0.04] [7]. The one-stage approach was also associated with fewer cases of clinical pancreatitis (2.4%) than the two-stage technique (8.4%) (OR 0.33; 95% CI 0.12–0.91; *p* = 0.03) [7]. In the current study, the rate of procedure-related complications was as low as 4% (*n* = 8) with 6 (3%) of patients experiencing post-interventional mild pancreatitis and 2 (1%) patients suffered a Strasberg type A bile leak. None of the patients required any additional invasive intervention and were discharged and fully recovered at follow-up. When comparing the two different approaches to CBD clearance in the current study, there was no statistical difference in procedural complications (Table 2).

Another disadvantage of the two-stage procedure is the timing of LC after ERCP. Recommendations vary from 72 h to a 6-week interval post-ERCP for LC, with a recurrent risk of CBD stones of 10% [16, 17]. Likewise, several investigators have noted a higher rate of conversion from LC to open surgery in patients subjected to a preoperative ERCP [18, 19]. One explanation offered by the authors of this finding is inflammation and scarring of the hepatoduodenal ligament through bacterial colonization due to the disruption of the sphincter of Oddi, making dissection of Calot’s triangle more challenging [19]. The mean operation time for LC + IO-ERCP at the two centers in this study was 105 (± 44) min, which should be considered a reasonable time for the combined procedure without any cases requiring conversion to open surgery. In comparison between the study sites, OUH had significantly longer procedure times at 91 (± 41) vs.120 (± 43) min.* (p* < 0.001), due to the use of the “rendezvous” approach and the  routine practice of intraoperative transcystic cholangiography.

Furthermore, other advantages with the one-stage approach include a single hospital admission and shorter hospital length of stay with a decrease in the total cost of care [17]. The cost of care for patients admitted to Orebro University Hospital in Sweden in the current study are outlined in Table 3. The median (LQ, UQ) postoperative length of stay was 2 (1,  3) days in the current study. The total hospital length of stay was 4 (3,  7) days. The total length of stay in the current study was influenced by factors such as patients with gallstone pancreatitis requiring observation until the laboratory values normalized before being cleared for surgery or older patients needing additional days in hospital to recover from surgery. Another advantage with the one-step procedure is patient satisfaction, which should be investigated further in future studies. Finally, the risk of patient drop-out due to compliance with two different hospital admissions is eliminated with the one-stage approach [20].

**Table 3:** Cost for care and procedures at Orebro University Hospital

	Cost (USD)
Total hospital care (mean ± SD)	9107 ± 3221
Laboratory tests (mean ± SD)	176 ± 107
Anesthesia (mean ± SD)	1199 ± 414
Operation (mean ± SD)	2925 ± 869
ERCP (mean ± SD)	1550 ± 404
Postop ICU (mean ± SD)	368 ± 404
Total ward stay (mean ± SD)	2473 ± 1987
Total ward stay per day (mean ± SD)	540 ± 126

Cost in USD calculated from SEK as of currency rate July 19, 2017 (1 USD = 8.29 SEK)

*USD* US Dollars, *SEK* Swedish Crowns, *ERCP* endoscopic retrograde cholangio-pancreatography, *ICU* intensive care unit

Many surgical centers do experience organizational and logistical obstacles with performing LC + IO-ERCP [8, 9]. This is mainly due to the fact that the procedure often requires collaboration between the surgical and gastroenterology/endoscopy teams. However, most of these patients are admitted to emergency surgery units in Europe rather than medical wards. With the evolving Trauma and Emergency Surgery subspecialty in Europe and Acute Care Surgery model in the United States, it may be appropriate to explore the possibility of endoscopy fellowships for surgeons, allowing the surgical teams to carry out both steps of the procedure. The current study does demonstrate low complication rates and did only include IO-ERCP cases carried out by surgeons. An alternative to this would be to have an ERCP-trained endoscopist on the emergency surgical service.

## Conclusion

Simultaneous laparoscopic cholecystectomy and intraoperative common bile duct clearance during the index admission is safe and feasible when performed by trained surgeons. Furthermore, cost-effectiveness and patient satisfaction warrant a prospective evaluation.
